# Liver Macrophages in the Pathogenesis of Viral Hepatitis

**DOI:** 10.3390/cimb48070687

**Published:** 2026-07-03

**Authors:** Ioannis Tsomidis, Angeliki Tsakou, Argyro Voumvouraki, Elias Kouroumalis

**Affiliations:** 1 Laboratory of Gastroenterology and Hepatology, University of Crete School of Medicine, Voutes Campus, 70013 Heraklion, Greece; itsomidi@gmail.com; 2 1st Department of Internal Medicine, AHEPA University Hospital, 54621 Thessaloniki, Greece; angtsak207@gmail.com (A.T.); iro_voum@yahoo.gr (A.V.); 3 Department of Gastroenterology, PAGNH University Hospital, University of Crete School of Medicine, 71500 Heraklion, Greece

**Keywords:** Kupffer cells, hepatitis B, hepatitis C, liver macrophages, liver immune tolerance, viral inflammation, viral persistence

## Abstract

Chronic hepatitis B virus (HBV) and hepatitis C virus (HCV) infection remain a world health problem leading to fibrosis and cirrhosis. Liver damage is primarily mediated by the innate and adaptive immune responses since HBV and HCV are not directly cytotoxic. Kupffer cells and liver-recruited macrophages are heavily implicated in both viral elimination and progression of the disease. HBV and HCV proteins polarize macrophages into either an M1 pro-inflammatory phenotype, promoting hepatocyte damage or into an M2 immunosuppressive phenotype, leading to viral persistence and fibrogenesis via cytokines such as interleukin-10 (IL-10) and transforming growth factor-beta (TGF-β). In this review a brief overview of the heterogeneity of liver macrophages in health and during chronic viral infection is presented. Recognition of viruses by macrophages and the modulation of macrophages by viral proteins in the pathogenesis of liver inflammation and injury are discussed in detail. Most importantly, the mechanisms that HBV and HCV are using to manipulate macrophages and escape elimination are also presented. The role of macrophages in the evolution of acute-on-chronic liver failure is analyzed. Finally, a concise presentation of the emerging, but not yet clinically used, therapeutic strategies targeting macrophages to control chronic HBV infection and restore the dysregulated immune response is discussed. In conclusion, this integrated review of liver macrophage implication summarizes the pathophysiology and pathogenesis of HBV and HCV including acute-on-chronic- liver failure and viral cirrhosis.

## 1. Introduction

The liver is a critical immune organ as it contains a considerable proportion of the total body macrophages. Approximately 80–90% of body resident macrophages are located in the liver, while liver macrophages constitute 20% to 35% of total liver non-parenchymal cells [1,2,3].

The majority of liver macrophages are localized in the periportal regions and do not directly contact hepatocytes. However, the resident macrophages defined as Kupffer cells (KCs) are in contact with the layer of liver sinusoidal endothelial cells (LSECs) that separates hepatocytes from the sinusoidal blood [4,5,6].

Liver macrophages are responsible for several important liver activities including the removal of aged or damaged red blood cells and aged platelets [7,8,9,10] and lipid metabolism [11]. Depletion of macrophages cause a significant reduction in serum iron levels [12,13]. Moreover, KCs phagocytose bacteria and debris reaching the liver through the portal or hepatic artery blood [14].

Above all, macrophages are important for their immune functions. They are capable of antigen presentation and recruitment of several types of immune cells [15]. In the normal liver, macrophages are involved in antigen processing, maintaining tolerance against gut-derived bacterial products. On the other hand, they may initiate inflammation through cytokine production, participating in liver damage, particularly in the evolution of viral infections. The liver is the target of several viruses, including HBV and HCV, which interfere with macrophage functions trying to evade elimination and establish viral persistence, leading to cirrhosis and possibly hepatocellular carcinoma, particularly after co-infection with the human immunodeficiency virus (HIV) [16].

Most studies of liver damage focus on the participation of effector T cells and natural killer cells (NKs) due to their ability to destroy hepatic cells. However, there is evidence demonstrating a central role for macrophages in inflammation of virally mediated liver damage [17]. In murine models of liver inflammation, pro-inflammatory cytokines produced by macrophages are both the initiators and effectors of inflammation and tissue damage, leading to fibrosis and dysregulated liver regeneration. These cytokines participate in disease progression by both damaging hepatocytes and by recruiting other effector immune cells in the liver. Experimental depletion of macrophages attenuates chronic inflammation [5,18].

Monocyte recruitment is tissue specific, allowing these cells to differentiate into either long-lived or short-lived macrophages [19,20,21]. In acute infections, the recruited monocyte-derived macrophages (BMDMs) are responsible for the pathogen clearance and the restoration of the KC population [22]. This is not always the case, as the macrophage pool is highly heterogeneous. Apart from the self-renewing embryo-derived KCs that populate the liver during fetal development, BMDMs may persist in the liver long after the clearance of the acute injury [5,23].

On the other hand, KCs are involved in the tolerant environment of the liver, which favors immune suppression, contrasting the macrophage-mediated inflammation. Tolerance against viral presence is mostly supported through IL-10 production from KCs [2,24]. KCs usually manifest an anti-inflammatory profile and phagocytose dying cells to avoid local inflammation [23]. However, this can compromise their pro-inflammatory capability, leading to exploitation by the viruses.

In this review, the dual effect of macrophages in the promotion of either tolerance and viral persistence or in the advancement of inflammation during HBV and HCV infection will be analyzed.

## 2. A Brief Overview of Heterogeneity of Liver Macrophages

Liver macrophages are either resident KCs or can originate from peripheral monocytes. KCs originate from yolk sac-derived erythromyeloid progenitors positive for colony-stimulating factor 1 receptor (CSF1R) that populate the fetal liver. Embryologically derived KCs (emKCs) have the ability of self-renewal and remain in the liver throughout adult life [25,26].

KCs communicate with hepatic stellate cells (HSCs), sinusoidal endothelial cells (LSECs) and hepatocytes, which constitute the KC niche. Aged and dying KCs release TNFα and IL-1, which activate LSECs and HSCs to transiently express monocyte chemoattractants. KC niche is transiently open and dead KCs are replaced by monocyte recruitment and their differentiation into monocyte-derived KCs (MoKCs), which proliferate to fill the niche. MoKCs are practically indistinguishable from emKCs [27,28].

### 2.1. Murine Liver Macrophages

Murine KCs express specific markers such as CLEC4F, VSIG4, CLEC2, and FOLR2 [29]. They also have several pattern recognition receptors (PRRs), such as toll-like receptors (TLRs), and retinoic acid-inducible gene I-like receptors (RLRs) [30].

On the other hand, BMDMs are recruited to the liver by chemokines, such as CCL2, and its receptors CCR2 [5]. BMDM recruitment initiation is mediated through activated TLR signaling in KCs or HSCs resulting in increased production of CCL2 [31,32]. Upon recruitment, BMDMs differentiate into several phenotypes depending on the local microenvironment.

The murine liver has two principal BMDM subsets identified by different Ly-6C expression levels as pro-inflammatory Ly-6C^high^ monocytes and patrolling Ly-6C^low^ monocytes [33]. Ly-6C^hi^ monocytes are characterized by their expression of inflammatory chemokine receptors, PRRs and production of inflammatory cytokines, whereas Ly-6C^low^ monocytes exhibit a higher expression of scavenging receptors [34]. It should be noted that a phenotypic plasticity exists between these subsets as Ly-6C^hi^ cells can change into restorative Ly-6C^low^ phenotype and vice versa [5]. Liver infiltration by Ly-6C^high^ cells during damage promotes liver fibrosis [35].

As mentioned before, two subsets of KCs populate the murine liver—bone marrow-derived KCs (moKCs), comprising approximately the third of the total KC population, and embryo-derived KCs (Em-KCs) [35,36,37,38]. Em-KCs have an excessive phagocytotic capability, whereas moKCs infiltrate the liver tissue after injury and initiate a pro-inflammatory response [39,40]. Thus, it was shown that EmKC were grossly reduced and substituted by moKC following acute fulminant viral hepatitis [41]. MoKCs can persist in the liver while BMDMs do not survive in the liver after resolution of inflammation [21]. EmKCs express two additional markers, namely MARCO and Tim4. Both emKCs and moKCs display an immunoregulatory gene signature possessing protective functions in the maintenance of liver homeostasis [42].

Murine KCs can also be separated in two discrete subsets according to the presence of the mannose receptor protein CD206 and the ESAM molecule. In accordance, KCs are further classified into two subpopulations, a prevalent CD206^low^ESAM- (KC1) and a CD206^high^ESAM+ (KC2). KC2 cells are implicated in the regulation of fatty acid metabolism, while KC2 with high expression of CD36 contribute to the regulation of the obesity-related oxidative stress [43]. CD36 deficiency attenuated the inflammatory response associated with acetaminophen (APAP)-induced acute liver injury [44]. On the other hand, deletion of total KCs by administration of clodronate liposomes in combination with deletion of TREM2+ macrophages worsen APAP-induced liver damage indicating that KC1 and TREM2 dependent macrophages have a protective role in APAP-induced liver injury by regulating necroptosis [45].

An additional subset of KCs has been identified. In irradiation-exhausted model, a population of radioresistant KCs was described, but no specific functions of these cells have been found [46].

### 2.2. Human Liver Macrophages

In the human liver, single-cell RNA sequencing (scRNA-seq) techniques have demonstrated that hepatic macrophages are also separated into several subpopulations [47,48,49,50]. In humans, blood monocytes are classified as classical (CD14^high^CD16−) and nonclassical (CD14−CD16^high^), which roughly correspond to the Ly-6C^high^ and Ly-6C^low^ peripheral monocytes in mice [34]. In the healthy human liver, in analogy to the murine liver, studies using single-cell technologies have identified resident KCs (CD68+MARCO+Timd4+) and BMDMs (CD68+MARCO-Timd4-) [47,51,52]. The CD68+MARCO+ Timd4+ cells correspond to emKCs participating in immune tolerance, whereas the CD68+MARCO- Timd4- cells correspond to the pro-inflammatory moKCs in mice [50]. An additional marker of Kupffer cells is CD163, which is a 130 kDa protein. The main function of CD163 is to remove hemoglobin–haptoglobin complexes from the circulation [53].

Moreover, the presence of CD32 was proposed as a good marker to discriminate between these population of macrophages. CD32^high^ macrophages seem to be involved in endocytosis and immune suppression, while CD32^low^ cells are implicated in inflammation and anti-microbial activity [54].

An additional discriminatory marker is CD49a, which is not expressed in moKCs. Moreover, emKCs produce the anti-inflammatory IL-10 cytokine, while moKCs do not produce IL-10 [55].

In certain inflammatory conditions, such as non-alcoholic steatohepatitis (NASH), macrophages express genes such as Spp1, Gpnmb (murine) and Trem2 and Cd9 (murine and human). These macrophages were designated as lipid-associated macrophages (LAMs) in mice, and scar-associated macrophages (SAMs) in humans [50,56,57]. LAMs were initially identified in adipose tissue [58].

Liver LAMs are similar to LAMs from adipose tissue. The number is small in the normal liver, but upon the onset of inflammation, LAMs comprise up to 50% of total liver macrophages [59,60].

They have also been referred as Trem2+ macrophages, as they express high levels of the triggering receptors expressed on myeloid cells 2 (Trem2) and glycoprotein non-metastatic melanoma protein B (Gpnmb), along with Cd9, Spp1, and Clec4d markers or as osteopontin (ssp1) +ve macrophages [61,62,63].

LAMs and SAMs have shown significant overlap [56] indicating that in fact these are equivalent populations. In addition, a protective subset of MerTK+ macrophages was described in human and murine acute liver failure, evolving during the resolution phase of the acute liver damage [64].

Single-cell RNA-Seq in liver biopsies from patients on HBV antiviral therapy led to the identification of an inflammatory, monocyte-derived macrophage population unique to inflamed livers that was designated as iMac [65].

It should be noted that in most studies of macrophages in liver diseases no differentiation of the macrophage subtypes involved was attempted. All CD68+ve cells were considered as macrophages, but no additional separation of macrophage subsets was reported despite the fact that both KCs and BMDMs are CD68+ve [16].

**Figure 1:**
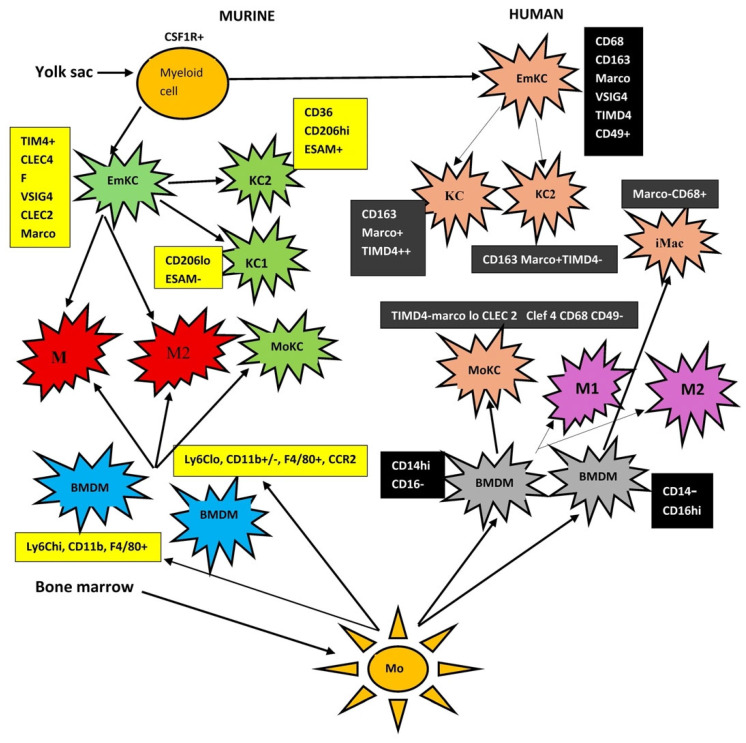
Ontogeny and heterogeneity of murine and human macrophages. Kupffer cells (KCs) originate from yolk sac through a CSF1R positive progenitor myeloid cell. Embryon-derived KCs (emKC) differentiate in KC1 and KC 2 cells in mice and KC1, KC2 and iMAC in humans. The last subset is found only in inflammation. On the other hand, recruited macrophages (BMDMs) originate from bone marrow monocytes. They differentiate in monocytderived KCs (moKCs), that are difficult to be distinguished from emKCs. Moreover, BMDMs are polarized in either M1 proinflammatory cells or in M2 anti-inflammatory cells. Polarization depends on the microenvironment stimulation. Yellow boxes indicate the murine macrophage markers, while black boxes indicate human markers. For further details see text.

Figure 1 presents the ontogeny and heterogeneity of murine and human Kupffer cells and bone marrow-derived macrophages.

CSF1R: colony-stimulating factor 1 receptor; EmKC/eMKC: embryonically derived Kupffer cell; KC: Kupffer cell; KC1: Kupffer cell subset 1; KC2: Kupffer cell subset 2; Mo: monocyte; BMDM: bone marrow-derived macrophage; moKC: monocyte-derived Kupffer cell; M1: classically activated macrophage; M2: alternatively activated macrophage; TIM4: T cell immunoglobulin and mucin domain-containing protein 4; VSIG4: V-set and immunoglobulin domain-containing 4; CLEC2: C-type lectin domain family 2; MARCO: macrophage receptor with collagenous structure; CD68: cluster of differentiation 68; CD163: cluster of differentiation 163; CD36: cluster of differentiation 36; CD206: cluster of differentiation 206; ESAM: endothelial cell-selective adhesion molecule; Ly6Chi: lymphocyte antigen 6 complex, high expression; Ly6Clo: lymphocyte antigen 6 complex, low expression; CD11b: integrin alpha M; F4/80: epidermal growth factor-like module-containing mucin-like hormone receptor-like 1; CCR2: C-C motif chemokine receptor 2; EmKCs: embryonic-derived KCs.

Table 1 summarizes the heterogeneity of murine and human liver macrophages.

### 2.3. Function of Liver Macrophages

The two important functions of liver macrophages are homeostatic tolerance and inflammatory response in liver disease as mentioned before.

Antigen presentation by KCs initiates CD4+ T cell arrest and proliferation of Foxp3(+) CD25(+) IL-10-producing regulatory T cells (Tregs) leading to immune tolerance [23].

In addition to their role in tolerance, KCs are mediators of inflammation that may be harmful to the liver. Thus, lipopolysaccharides (LPSs) may induce KCs to secrete damaging inflammatory cytokines such as tumor necrosis factor (TNF)-α, interleukin (IL)-1β, and IL-6 leading the hepatocytes to apoptosis and necrosis [67,68]. Activation of the T cell co-stimulatory receptor 4-1BB (CD137) present in liver KCs and BMDMs is essential to initiate liver inflammation and hepatitis through production of IL-27. Treg cell removal promotes 4-1BB agonist-induced hepatitis. Interestingly, administration of CTLA-4 checkpoint inhibitors attenuates hepatitis, while PD-1 inhibition exacerbates it. Loss of the chemokine receptor CCR2 blocks 4-1BB agonist hepatitis [69].

KC are also able to activate NK cells and natural killer T (NKT) cells, [70]. Moreover, KCs express cytotoxic molecules such as TRAIL, Fas-ligand, granzyme B, perforin, and reactive oxygen species (ROS), which may damage hepatocytes [71,72]. KCs may also initiate liver inflammation through the cGAS-STING pathway. The expression of cyclic GMP-AMP synthase (cGAS) and Stimulator of Interferon Genes (STING) are low in hepatocytes but are significantly greater in KCs and play an important role in the detection of cytoplasmic DNA [73]. STING is directly activated by cyclic guanosine monophosphate–adenosine monophosphate (cGAMP), a second messenger produced by the cGAS [74].

The multi-faceted function of liver macrophages in inflammation and immunity was better studied when two distinct functional subtypes were described. M1 and M2 macrophages were classified according to differences in their metabolism and function [75]. M1 macrophages maintain an increased glycolytic activity and a low oxidative phosphorylation (OXPHOS), while M2 macrophages have high OXPHOS and low glycolytic activity [76]. Importantly, those polarized cells may interchange phenotypes depending on the microenvironment [77].

M1 macrophages are polarized by stimulation with IFN-γ, TNFα, GM-CSF, and TLR ligands [78]. They produce pro-inflammatory cytokines inducing the transformation of HSCs into myofibroblasts [79]. Μ1 macrophages phagocytose damaged cells and produce increased amounts of reactive ROS and reactive nitrogen species (RNS) [80].

M2 macrophages produce immunosuppressive mediators such as IL-10, TGF-β, IL-4 and IL-13 [81]. Among these cytokines, TGF-β plays a crucial role in HSCs activation and liver fibrosis [82].

M2 macrophages can be further sub-classified into distinct phenotypes based on the stimuli of their induction. M2a is induced by IL-4 and IL-13, M2b by immune complexes, M2c is stimulated by IL-10, TGF-β and glucocorticoids and M2d is induced by IL-6, TLR ligands and adenosine [83,84]. M2a and M2b macrophages produce pro-fibrotic factors including TGF-β and insulin-like growth factor (IGF). M2c cells inhibit inflammation and fibrosis and induce T reg cells. M2d macrophages are phenotypically similar to tumor-associated macrophages (TAMs) contributing to angiogenesis and metastasis [83,84].

In early liver damage irrespective of etiology KCs and BMDMs express an M1 phenotype that promotes inflammation through NLRP3 inflammasome activation followed by HSC transformation as mentioned before. A switch toward M2 phenotype happens during fibrosis progression mediated by IL-4 and IL-13 liberated from Th2 cells and apoptotic hepatocytes [85,86]. M2 macrophages were believed to promote fibrosis reversion by producing matrix-degrading metalloproteases [87] and anti-inflammatory IL-10 [88,89]. However, M2 cells may also favor fibrosis through galectin-3-mediated HSC transformation [90]. Similarly, other macrophages such as TREM2+LAMs may demonstrate both pro- and anti-fibrotic behavior [58,91].

The older M1/M2 classification is now considered obsolete. Several different macrophage subpopulations have been identified in the fibrotic liver. Interestingly, subsets expressing both M1 and M2 markers have been described [92,93,94]. Moreover, disease-specific subsets have been described that do not fit to the M1/M2 classification such as CX3CR1+ restorative macrophages in viral hepatitis [32,95,96,97]. In a mice model of Concanavalin A-induced hepatitis, the deficiency of CX3CR1 increases pro-inflammatory cytokine production in macrophages and T cells by enhancing the phosphorylation of NF-κB p65, which exacerbates liver injury [97].

While M1 or M2 phenotypes are still widely used [98] the observed overlaps suggest more complex responses and phenotypes [99,100]. However, this classification is useful for two main reasons. First the functions of M subtypes allow for a more or less clear distinction in different studies. Therefore, a model of communication and comparisons between different studies is available. Moreover, the role and function of the majority of recently described subtypes have not been adequately studied so far.

Similarly, the cytokine IL-10 is generally considered as an anti-inflammatory cytokine that can repress T cell responses and release of IL-2, IFN-γ, and TNF-α [101]. But IL-10 can also have immunostimulatory effects including expansion of CD8+ T cells [102].

A useful marker which is associated with liver damage and macrophage activation is CD163 that is shed from the surface of macrophages upon TLR activation [103].

To make things more complicated, recent research identified a BMDM population unique to viral liver inflammation and distinct from KCs, with an inflammatory profile designated as IMAc as mentioned before. Further analysis revealed that the differentiated iMacs represented a unique state between M1 and M2 polarization. They expressed M1 markers, such as CD40, but lacked CD86, and expressed the M2 marker CD16, but lacked CD206 and CD209. At the functional level, iMacs were largely inflammatory-producing cytokines such as IL-1β, IFN-α2, MCP1, IL-8 and IL-23, but they also secreted IL-10. iMacs differed from M1 macrophages by their reduced production of TNF-α, IL-6, and IL-12p70 upon TLR stimulation. iMacs retain their inflammatory profile long after the resolution of inflammation. In fact, they were identifiable even after 4 years of antiviral therapy [65].

Reviews on KC ontogeny, heterogeneity and function have recently been published [16,21,46,103,104].

## 3. The Role of Liver Macrophages in HBV

KCs and BMDMs act like a double-edged sword in HBV infection. First, they participate in the anti-HBV immune response to promote viral elimination. On the other hand, they have a prominent role in HBV viral persistence. Therefore, it is not surprising that liver macrophages are used by HBV to promote its replication [105].

### 3.1. Recognition of HBV by Macrophages

Viral components could be present after phagocytosis and viral epitopes could be recognized by immune receptors in liver macrophages. In vitro studies demonstrated that HBV antigens such as HBV envelop proteins and HBV core antigen (HBc) were able to induce cytokine secretion in KCs and BMDMs. An early nuclear factor kappa-B (NF-kB)-dependent production of inflammatory mediators in primary human KCs incubated with HBV has been reported [21]. It was also shown that KCs and BMDMs interact with the HBV surface antigen (HBsAg) in vivo and in vitro. HBsAg induced pro-inflammatory cytokine production by KCs and BMDMs followed by NK cell activation leading to either viral elimination or liver damage and persistence of the virus [21]. Moreover, in liver biopsies from HBV patients, IL-23 and IL-23R were significantly upregulated. In vitro differentiated macrophages of healthy donors revealed that HBsAg induces IL-23 secretion through interaction with a mannose receptor [21].

HBcAg can be recognized by both TLR2 [21,106] and heparan sulfate proteoglycan (HSPG) on macrophages through its arginine-rich domain. Recognition leads to an effective production of pro-inflammatory molecules. In a murine model of high HBV replication rate, a TLR3-mediated interferon response from non-parenchymal sinusoidal cells was also observed [107]. HBcAg was also demonstrated to bind to peripheral mononuclear cells (PBMCs) and initiate the release of IL-18 [108].

HBV components were identified in the liver of viremic patients, mostly in hepatocytes, but also within Kupffer cells. Interestingly, it was the KC1 cluster that showed higher amounts of HBV mRNA in comparison to other sinusoidal cells with approximately 26% of KC1 manifesting measurable HBV mRNA levels. These findings indicate that KCs directly phagocytose infected hepatocytes as mRNA contamination from dying hepatocytes is not a probable explanation [109].

HBV functions as a stealth virus and does not interfere with the immunity system of hepatocytes. On the other hand, macrophages require exposure to high HBV levels to be able to sense the virus. This may explain the long period between acute infection and HBV persistence leading to chronic infection [110]. It was recently shown that the complete HBV virion can infect macrophages. HBV appeared in liver macrophages as early as one hour after infection but it was detected in hepatocytes only after 16 hours post-infection. HBV bound to apolipoprotein-A (Apo-E)-rich lipoproteins in human serum entered liver macrophages through the receptor-mediated lipoprotein endocytic pathway. Within liver macrophages, HBV was transported and accumulated to recycling endosomes by using the transport system of lipoproteins. Thus, HBV avoided lysosomal degradation. Furthermore, HBV returned to cellular surface and was transported to hepatocytes by taking over the reverse cholesterol transport pathway. The exact mechanism of HBV association with Apo-E lipoproteins has not been clarified [111,112].

A partial explanation may be provided by recent findings. C1q which is the inducer protein of the classical complement cascade is mostly produced by KCs as an inactive form along with ApoE. ApoE binds with high affinity only to the activated form of C1q. Viral particles may activate C1q leading to the formation of the C1q-ApoE complex, which in turn is the scaffold for HBV endocytosis [113].

On the other hand, involvement of macrophages with ApoE may in fact lead to a viral elimination mechanism. Continuous HBV stimulation initiates a dysregulated aberrant lipid accumulation that recruits and activates a specific cluster of macrophages, possibly TREM2+LAMs, producing high levels of IL-1β, IL-6, IL-12 and TNF-α followed by recruitment of other immune cells and elimination of the virus [114].

New techniques such as single-cell RNA sequencing and spatial transcriptomics have provided new data on the composition of immune cells in viral hepatitis. The new technologies provide an in-depth understanding of cellular and molecular dynamics across various states of healthy and diseased livers. Through the integration of sophisticated bioinformatics strategies, they allow for detailed exploration of cellular heterogeneity, transitions in cell states, and intricate cell–cell interactions with remarkable precision [115].

Thus, increased levels of intrahepatic exhausted CD8+ T (Tex) cells were found in patients with HBV, both in the immune active (IA) and the acute recovery phase (AR) but the origin was different. In IA, Tex cells were mostly originated from liver-resident GZMK+ve effector memory T cells. By contrast, peripheral CX3CR1+ve effector T cells and GZMK+ve effector memory T cells were the main source of Tex cells in AR. Importantly, in IA but not AR, significant cell–cell interactions were found between Tex cells and regulatory CD4+ T cells, as well as between Tex and FCGR3A+ve macrophages [116].

Moreover, the intrahepatic molecular signatures in both patients with active chronic hepatitis B and those with functional cure was different from those found in matched PBMCs. Functional cure is associated with altered adaptive immune response characterized by CD4 cytotoxic T lymphocytes and an activated innate response represented by liver-resident natural killer cells and specific Kupffer cell subtypes [117].

On the other hand, high-resolution spatial transcriptomes in liver biopsies demonstrated that transcriptionally active HBV integration occurred in chronically HBV-infected patients at different phases, including patients with HBsAg loss. Antiviral treatment was associated with a decreased number and extent of transcriptionally active viral integrations [118].

However, the new techniques are mostly descriptive, while an association with functional characteristics need further extensive studies.

### 3.2. Liver Macrophages in HBV-Disease Pathogenesis

Liver injury induced by macrophages is mediated by the secretion of inflammatory cytokines as mentioned before. The resultant inflammation is in fact an antiviral mechanism trying to eliminate the virus which operates against many HBV genotypes. The resultant tissue damage is a collateral effect. Cytidine deaminase is involved in this effort to restrict viral invasion [119]. Macrophage elimination can mitigate the inflammatory response in experimental animals [5,18]. TNF-α and IL-1β, which can damage hepatocytes can also activate HSCs, promoting fibrosis and oncogenic pathways [120,121].

Liver macrophages may also be involved in the elimination of HBV through interference with the function of T lymphocytes. The pro-inflammatory mediators that are produced by macrophages induce specific immune responses in addition to the direct inhibition of HBV proliferation. Thus, IL-12 enhances HBV-specific CD 8+ T and CD4+ T cell effects [67], while IL-18 shifts the balance between Th1 and Th2 toward Th1 during the antiviral response as it enhances the response of Th1 to viral infection. However, functional polymorphisms may favor the replication of the virus [122].

Dysfunctional HBV-specific CD8+ T cells are present in chronic HBV. CD4+ T cells permit KCs to secrete IL-12 and IL-27. In turn, IL-12 amplifies the CD4+ T cell pool, while IL-27 is necessary for CD8+ T cell restoration of functions as shown in murine models and isolated cells from HBV patients [123]. Moreover, increased IL-23 production by KCs is critical for HBsAg-mediated differentiation of naive CD4(+) T cells into Th17 cells leading to liver damage through the IL-23/IL-17 axis [124].

In HBV-related acute hepatitis, the number of KCs is decreased, but the remaining cells can still initiate an antiviral response [125]. Em-KCs that are CD206+ESAM+ react to IL12 and present antigens to promote T cell-mediated HBV destruction [126]. KCs also recognize HBV and enhance NK cell activation via IL18 [70]. In addition to pro-inflammatory cytokines, another subset of KCs is producing factors, such as ROS and Fas-ligand [71], TNF-related apoptosis-inducing ligand (TRAIL), granzyme B and perforin, that destroy hepatocytes by apoptosis or necrosis [127]. Elevated concentrations of Fas-ligand, TRAIL, and TNF-α were detected in chronic HBV. Moreover, the macrophage activation marker soluble CD163 was increased during liver injury irrespective of the stage of HBV infection. sCD163 levels significantly diminished after 12 weeks of Tenofovir treatment [17].

A recent report demonstrated the significance of the gp96 protein in the pathophysiology of chronic HBV. Serum gp96 levels were increased in patients with chronic HBV (CHB) and acute-on-chronic liver failure (ACLF). The gp96 level positively correlated with hepatic necro-inflammation. The exposure of KCs to gp96 induced the secretion of pro-inflammatory cytokines indicated that gp96 released from necrotic hepatocytes aggravates liver damage and possibly participates in liver failure mainly by activating KCs [128].

KCs and BMDMs are also interfering with the equilibrium between M1 and M2 polarization in HBV. M1 macrophages prevailed in the liver of CHB patients and were co-localized with activated HSCs in fibrotic areas. At the same time, the M2 marker IL-10 was repressed. HBeAg was the driver of M1 polarization. In vitro experiments showed that HBV switched a macrophage cell line towards an M1 phenotype that activated an HSC cell line indicating that pro-inflammatory M1 macrophages collaborate with HSCs in fibrogenesis [129].

Incubation of M1 macrophages with either HBV or with the individual viral proteins induced the secretion of IL-1β, which has a strong HBV-suppressive effect. HBsAg binds to CD14, the co-factor of TLR4 [130], and both HBeAg and HBcAg [131] bind to TLR2. HBV also upregulated the expression of peroxisome proliferator-activated receptor α (PPARα) and forkhead box O3 (FOXO3) in hepatocytes leading to increased HBV transcription. This upregulation was inhibited by IL-1β, which also diminished HBsAg and HBV core protein levels leading to reduced HBV replication. However, HBV can reprogram M1 macrophages to enhance OXPHOS and repress the production of IL-1β leading to HBV persistence [132].

These findings are in accordance with an earlier report of HBeAg-negative CHB patients, where increased alanine aminotransferase was associated with increased M1 macrophages in the liver, when compared with the immune tolerant group. Increased levels of M1 macrophages allow patients to combat HBV infection [133].

An interesting observation was done on HBV-negative mice born to HBV-positive mothers. They responded with a KC M2-like anti-inflammatory polarization when they were exposed to HBV in the presence of HBeAg. In contrast, KCs isolated from control mice that were born to HBV-negative mothers would undergo M1-like pro-inflammatory polarization under the same experimental conditions. These results demonstrated that HBV could initiate either M1-like or M2-like KC polarization depending on the previous exposition of KCs during pregnancy [134].

The reason for these discrepancies is not clear. It seems that HBeAg may in fact drive macrophages to either M1 or M2 polarization. Possible explanations include the different experimental conditions and the different models that were used. An alternative explanation could be that it is the complete HBV virion that is mostly responsible for the macrophage polarization and not the individual proteins.

In another clinical study, serum from cured patients with HBV infection induced more M1 cells than that from uncured patients. Patients had significantly lower proportions of CD86+ M1 and CD206+ M2 macrophages in their livers than healthy controls. M1 polarization of intrahepatic KCs increased HBsAg loss by upregulating the effector capability of tissue-resident memory T cells [135].

M1 polarization in HBV is promoted by several other mechanisms. Hepcidin overexpression in a mouse model of HBV infection demonstrated that iron accumulation was responsible for the polarization of M1 macrophages via the hepcidin–ferroportin axis, after activation of the IL-6/JAK2/STAT3 signaling pathway [136]. HBV dsDNA also upregulated M1 polarization by activating the cGAS-STING pathway, while no significant M2 polarization was observed [137]. Interestingly, the activation of STING signaling in a murine model diminished HBV replication through epigenetic repression of ccc DNA. Moreover, it may ameliorate HBV-initiated liver fibrosis through the repression of macrophage inflammasome activation [138].

However, the situation in chronic HBV infection is not clear. Multiple macrophage populations are found in the liver during active damage in patients with CHB.

Inflammatory KCs and inflammatory BMDMs were recently described. These cells were designated as iMacs as mentioned before. iMacs were unique compared to macrophages found in either healthy or cirrhotic liver as they are confined to patients with active inflammation, but after resolution of the inflammation they persist in the liver for a long time. KCs seem to participate in iMac differentiation. KCs are not eliminated and survive during liver inflammation. Different subsets of KCs control different pathways of inflammation in patients with CHB. A discrete subpopulation of KCs controls monocyte recruitment through CXCL12, while a different subpopulation seems to control iMacs differentiation [65].

Important data on the pathogenesis of liver damage during HBV infection were collected from studies on patients with elimination of serum HBsAg and functional cure (FC) [139]. Previous studies have demonstrated the important role for virus-specific T cells as the main cell responsible for FC [140]. However, the role of KCs and NK cells may be equally important [126,141,142].

Increased numbers of NK cells in FC expressing an increased amount of activating receptors such as CD38, NKG2D and TRAIL and low levels of inhibitory receptors such as PD-1 and Tim-3 were recently described [117]. This was accompanied by increased numbers of pro-inflammatory CD14+ve monocytes and FOLR2/VSIG4 KCs in FC compared to CHB [117]. FOLR2/VSIG4 KC gene profile was similar to the KC2 subtype which is capable of antigen presentation and restoration of exhausted T cells in murine models of HBV infection [126].

The above-mentioned macrophage responses were age-related and this may explain the difference in prognosis of HBV infection according to age. It is well known that almost 90% of infected neonates do not clear HBV and develop chronicity, whereas approximately 30% of children aged 1–5 years old and 95% of adults exposed lead to elimination of the virus [143]. Age-dependent HBV immune response was also demonstrated in a murine model [144,145]. The maturation of BMDMs and KCs is the decisive factor that controls age-related HBV elimination [125,144]. The 12-week-old mice had increased TNFα-secreting Ly6C+ monocytes and reduced IL-10-secreting KCs compared to the 6-week-old mice [125]. Reduction in KCs by clodronate enhanced HBV elimination in previously HBV-tolerant mice [125,134,146] due to increased Ly6C+ monocyte recruitment [125].

Gut microbiota may also be implicated in the age dependency of HBV clearance. KCs originating from germ-free mice exhibited a phenotype favoring tolerance. The maturation of KCs correlated with the density of gut-derived microbial-associated molecular patterns (MAMPs). Therefore, HBV is benefited by the immaturity of liver macrophages and the instability of gut microbiota in early life to avoid elimination [147].

### 3.3. Tolerance and Persistence of HBV Infection

#### 3.3.1. Tolerance and Liver Macrophages

A strong initial immune response is important to eliminate infections. HBV-specific CD8+ T cells either directly attack and eliminate infected cells or repress viral replication via non-cytopathic interferon-mediated pathways. Effector CD8+ T cells are activated in the early stages of infection, but gradually lose their function during chronic viral infection, a phenomenon known as exhaustion. HBV has several pathways in the attempt to evade immune clearance and establish chronic disease. Liver macrophages, particularly KCs, are the most important mediators in immune evasion and the induction of tolerance [148].

KCs repress effector CD8+ T cells, expand the pool of Tregs, and phagocytose debris and microbial products without inducing a strong inflammatory response [149,150]. Moreover, during phagocytosis, they secrete immunosuppressive factors such as TGF-β and IL-10 establishing local immunosuppression that restricts lymphocyte activation and protects liver homeostasis [151]. During CHB, KCs maintain a similar phenotype with high IL-10 and TGF-β production that favors viral persistence suppressing antiviral T cell activity [132,152].

Earlier studies have clarified mechanisms for the maintenance of tolerance despite the presence of HBV. CD68+/CD86+ macrophages were significantly increased in comparison to the number of CD68+/ CD80+ macrophages in the liver of patients with CHB [153]. The upregulated ratio of CD86+/CD80+ macrophages shifts the balance of T helper cells toward a T type 2 (Th2) response, which favors tolerance. The Th2 response leads to increased production of pro-fibrotic mediators such as TGF-β, platelet-derived growth factor (PDGF) and Galactin-3, promoting therefore the progression of liver fibrosis [116,154,155,156].

The mechanisms of CD8+ T cell dysregulation during CHB were also studied. Hepatocytes or KCs were infected in mice. These studies proved that priming of CD8+ T cells by infected hepatocytes led to defective CD8+ T cells with dysregulated antiviral activity. On the other hand, priming by infected KCs led to effector CD8+ T cells able to mount a potent antiviral response [157]. KC-primed CD8+ T cells demonstrated increased expression of IL-2, the critical T-cell growth factor [157,158]. The ability of IL-2 treatment to restore dysfunctional CD8+ T cells during HBV infection is mediated by the KC2 subset. KC2s by sensing IL-2 and presentation of hepatocellular antigens, override the tolerogenic potential of the hepatic microenvironment [126].

In patients with CHB, liver macrophages produced anti-inflammatory mediators such as IL-10, and TGF-β, while production of IL-1β, IL-6, IL-12, and TNF-α was downregulated [138,159,160,161]. In addition, healthy BMDMs were polarized to an anti-inflammatory phenotype when incubated with HBV antigens derived from chronic HBV patients [132,162].

#### 3.3.2. Implication of TLRs in Tolerance and Persistence

Tolerance against HBV is also mediated through the implication of TLRs. Reduction in TLR2, TLR3 and TLR4 numbers on the surface of KCs and macrophages have been reported in HBeAg+ve patients [163,164].

The most common mechanism though, is the downregulation of the effects of TLRs in immune cells. Decreased TLR2-mediated activation of c-Jun N-terminal kinases (JNKs) with decreased secretion of IL-6, TNF-α and IL-12 were found in monocytes during co-incubation with HBsAg and HBeAg [165]. It was suggested therefore, that viral proteins through TLRs decreased the surveillance competence of HBV-infected KCs leading to immune escape.

TLR2 signaling has also a dual role in HBV. During the early phase of HBV secretion of inflammatory cytokines is increased, which restricts HBV transcription. However, IL10 secretion is also initiated superseding the anti-HBV effect [166,167]. In that respect the term stealth virus may be not relevant as HBV infection initiates early cellular responses but the final outcome is elusive. TLR2 signaling may initially arrest, but finally permits HBV persistent transcription in hepatocytes [168].

Other findings contradict these reports. A genetic deficiency in TLR2 improved HBV elimination, whereas activating TLR2 led to more stable HBV persistence, suggesting that TLR2 activation is critical in HBV persistence. In this study, TLR2 activation also caused CD8(+) T cell exhaustion in HBV-carrier mice. TLR2 expression on KCs was increased in HBV-infected mice, leading to HBV persistence. Moreover, after KC depletion, CD8(+) T cells were activated in HBV-infected mice, leading to HBV elimination. KCs secreted increased amounts of IL-10 upon TLR2 activation by HBcAg stimulation further inhibiting CD8(+) T cell function. It was suggested, therefore, that KCs sustain liver tolerance by inducing specific CD8(+) T cell exhaustion via IL-10 production after TLR2 activation by HBcAg stimulation in contrast to the previous reports [169]. The reason for this discrepancy is not clear, but differences in experimental conditions may be a logical explanation as IL-10 expression may be different in different experiments. Furthermore, direct comparisons cannot be made as there are many parameters that may influence the results. Data are provided by either in vitro models or clinical studies. Incubation times vary in experimental conditions, while disease stage, viral loads and different genotypes may play a role in clinical studies.

As mentioned before, macrophages sense HBV infection via PRRs such as TLR3, TLR4, and cytosolic DNA sensors, initiating antiviral responses including the pathway of STING–TANK-binding kinase 1 (TBK1)–Interferon regulatory factor 3 (IRF3) and NLRP3 inflammasome signaling [170,171,172]. However, HBV proteins can modulate these antiviral responses leading to inhibition of this pathway and immune escape [173].

Interestingly, the TLR8 agonist Selgantolimod (SLGN) upregulated KCs monocyte markers such as S100A12 and decreased genes associated with the KC identity such as SPIC. Hepatocytes treated with SLGN decreased the HBV entry NTCP receptor and impaired HBV entry. Co-treatment with an IL-6 neutralizing antibody reversed the inhibition of HBV entry. It was suggested therefore, that TLR8 activated KCs produce IL- 6 that directs hepatocytes to downregulate NTCP levels, impairing their susceptibility to HBV infection [174].

#### 3.3.3. IL-10 Production and M1/M2 Balance

Anti-inflammatory cytokines such as IL-10 and TGF-β [142,175,176] are produced by KCs, contributing to HBV immune evasion and fibrotic remodeling. As mentioned before, HBV may escape immune elimination by modifying macrophage polarization [46,177].

In CHB patients, M2 type macrophages with immunosuppressive effects outnumber M1 cells which are inhibited by HBV. This finding was associated with increased expression of IL-10. Mechanistically, HBsAg and HBeAg act through the SIRT1/ Notch1 pathway, leading to decreased NF-κB nuclear translocation in macrophages. These changes contributed to M2 polarization [178]. Increased M2 macrophages was found in patients with CHB or HBV patients with acute liver failure, indicating that M2 macrophages might act as immune suppressors [154,179]. This suggestion has been confirmed by several studies. CD163 is highly expressed in M2 macrophages and is commonly used as a marker for the M2 phenotype [180]. It was reported that CD163+ macrophages were significantly expanded in liver tissue from patients with CHB [142]. The levels of serum CD163 were reduced after successful antiviral treatment leading to HBsAg loss [181]. In an HBV-infected murine model, CD163 deficiency enhanced the clearance of HBsAg, HBeAg, HBV DNA, and HBcAg. Moreover, CD163 deficiency upregulated the appearance of HBV-specific effector T cells. Furthermore, CD163 deficiency reduced KCs-derived IL-10 secretion [182].

Interestingly, dysbiosis of the gut microbiota in a murine model led to endotoxemia with KC IL-10 production that promoted T cell suppression and tolerance against HBV [183].

Studies comparing MARCO+ and MARCO− KCs in HBV are few. MARCO+ KCs are tolerogenic subsets, supporting HBV persistence. They phagocytose apoptotic hepatocytes and produce IL-10 to restrict strong inflammatory response that might clear the virus. In contrast, MARCO− KCs elicit intense inflammation via cytokines such as TNF-α which lead to viral clearance at the expense of liver damage if left unchecked [184].

Macrophages also display GABAergic signaling mechanism. Stimulation of type A GABA receptors favors M2-polarization, thus increasing HBV transcription, but the vulnerability of hepatocytes to HBV infection is not altered [185,186].

#### 3.3.4. Implications of HBV Proteins in Tolerance and Persistence

As already mentioned, HBV components modulate several macrophage signaling pathways [187]. In particular, the viral polymerase and HBx prevent interferon production by interfering with the activation of interferon regulatory factors IRF3 and IRF7, which are essential for triggering antiviral responses. HBx also impairs mitochondrial antiviral signaling and interferes with RIG-I-like receptor pathways, further inhibiting antiviral signaling [188].

HBeAg represses NLRP3 inflammasome activation and IL-1β secretion either by inhibiting NF-κB phosphorylation or by inhibiting caspase-1 through suppression of ROS production [159]. In addition, HBV polymerase is attached to STING preventing STING-dependent cytosolic DNA sensing and type I interferon production [189]. These effects in parallel favor HBV persistence. However, other studies revealed that HBeAg acts as a double-edged sword. Incubation of macrophages with recombinant HBeAg increased production of TNF-α and IL-6 in macrophages triggering an inflammatory response. Data suggests therefore, that HBeAg can both initiate tolerance in macrophages and induce a strong inflammatory response. The final response depends on conditions that have not been identified at present [190].

HBV utilizes escape methods on the cellular surface in addition to the previously described intracellular pathways. HBsAg has been reported to impair TLR4 and TLR2 downstream signaling. It specifically disrupts TLR2 ligand-induced IL-12 production by impairing JNK activation [191,192]. Production of pro-inflammatory M1 cytokines was disrupted by HBsAg in PBMCs, but the production of IL-10 was not impaired [193]. Similar results were reported in a macrophage cell line where HBsAg significantly suppressed M1 cytokines [165,193,194].

HBcAg favors virus elimination as shown in HBV-transfected mice. Absence of HBcAg resulted in decreased recruitment of infiltrating TNF-α+ Ly6C+ monocytes compared to control transfection, leading to prolonged HBV infection [125]. HBcAg had a very small effect on M1 polarization, but it diminished M2 polarization promoting their secretion of IL-6 and TNF-a. Moreover, M2 macrophage stimulated with HBcAg regained their competence to activate CD8+T cells with increased production of IFN-γ. Clinical observations confirmed a permissive role of HBcAg in the creation of an inflammatory response changing the cytokine profile produced by M2 macrophages. Increased levels of pro-inflammation cytokines in M2 macrophages from CHB patients upon HBcAg stimulation were observed [131]. However, HBcAg may promote HBV persistence by inducing increased expression of programmed death-1 (PD-1) on CD4+ T cells accompanied by an increase in PD-L1 expression on macrophages, leading to exhaustion of T cells [195].

The conflicting results on the dual role of HBV proteins may be explained in part by examining the metabolism of liver macrophages. As analyzed before in Section 2.3, macrophages can undergo M1 polarization with low oxidative phosphorylation (OXPHOS) and high glycolytic activities. In an experimental study, M1 macrophages stimulated by HBV overproduced IL-1β leading to an HBV repressive effect. HBV interfered with the production of IL-1β causing a high OXPHOS activity distinct from that of conventional M1-like macrophages. OXPHOS downregulated the expression of IL-1β leading to increased expression of PPARα and FOXO3 in hepatocytes that favors HBV replication. This atypical metabolism was mediated by HBeAg, which initiated death receptor 5 (DR5) via TLR4 to induce death-associated protein 3 (DAP3). DAP3 then promoted OXPHOS by triggering mitochondrial genes. HBeAg also upregulated glutaminases increasing glutamate, which is converted to α-ketoglutarate ultimately leading to OXPHOS promotion through the tricarboxylic acid cycle. These results indicate that HBV can reprogram the metabolism of macrophages to increase OXPHOS and curtail the antiviral response [132]. HBV also initiated hyperacetylation of critical enzymes of metabolism such as citrate synthase and pyruvate dehydrogenase leading to disruption of the tricarboxylic acid cycle that switched macrophages toward an M2 immunosuppressive phenotype that favors HBV persistence [196].

#### 3.3.5. Macrophage Immune Checkpoints and Virus Persistence

Programmed death ligand 1 (PD-L1) checkpoint negatively controls the immune response by binding to its receptor programmed death-1 (PD-1). Evidence has shown that increased expression of PD-L1 is accompanied by increased liver infiltration with M2 macrophages. Mechanistically, PD-L1 binding to PD-1 shifts CD206 macrophages to M2 polarization and metabolic reprogramming via Erk/Akt/mTOR [197,198]. PD-L1 expression in macrophages is a major inhibitor of antiviral immunity in CHB [199]. HBV upregulates PD-L1 expression on macrophages driving T cell exhaustion after binding to PD-1 of T cells. Moreover, PD -L1 repression in liver promotes efficiency of therapeutic vaccination for CHB [200]. Importantly, HBV activation of phosphatase and tensin homolog (PTEN) signaling also increases PD-L1 expression promoting HBV immune evasion [201]. PD-L1 is constitutively expressed on LSECs, KCs and HSCs [202]. After the elimination of the initiating antigen, PD-1 expression eventually declines, but if T cells are exposed to antigens for over two weeks, T cell exhaustion becomes permanent, and these cells do not recover through the removal of antigen exposure [203].

A combination of molecules that prevent interaction between PD-1 and its ligand and activate CD137 signaling increased responses of intrahepatic HBV-specific T cells [204].

Other checkpoint molecules are also involved in HBV persistence. After HBV infection, an increased expression of Fas was identified in hepatocytes, while the expression of FasL was upregulated in KCs [205]. The Fas/FasL system has also a dual role in liver immunology. FasL expressed by KCs may bind to Fas of infected hepatocytes leading to apoptosis and elimination of HBV [206]. However, Fas is also expressed on lymphocytes, and a parallel apoptosis of lymphocytes can promote immune tolerance and persistence of HBV [207].

Finally, an increase in galectin-9 on liver macrophages associated with an increase in HBV-specific Tim-3+ CD8 T cell was reported in CHB. This Tim-3/galectin-9 interaction will eventually lead to T cell exhaustion and HBV persistence [208].

**Figure 2:**
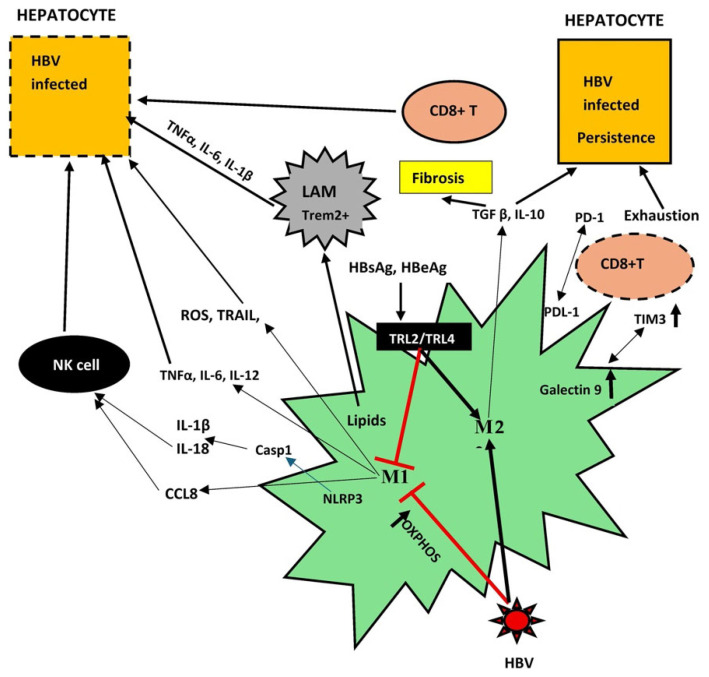
The role of macrophages in HBV inflammation and persistence. M1 macrophages and Kupffer cells recognize HBV proteins such as HBsAg and HBeAg and secrete pro-inflammatory cytokines such as TNFα, IL-6, IL-1β through activation of the NLRP3 inflammasome. They also produce molecules such as ROS, perforin and TRAIL that may lead to apoptosis or direct hepatocyte damage. Moreover, they recruit cytotoxic cells such as NK cells and CD8+ T lymphocytes by secretion of chemokines (not shown for clarity). Accumulation of lipids in macrophages leads to differentiation into pro-inflammatory lipid-associated macrophages (LAMs) enhancing damage of hepatocytes. On the other hand, complete HBV virion recognition blocks M1 polarization switching into M2 phenotype. A similar change is caused by recognition of viral proteins through TLR2/TLR4 receptors. M2 macrophages induce a tolerogenic environment through production of IL-10 and TGF-β that favors HBV persistence and development of fibrosis. Additionally, upregulation of galectin-9 and PDL-1 in macrophages lead to CD8+ T cell exhaustion and persistence of the virus. For further details see text.

Figure 2 diagrammatically presents the role of macrophages in HBV elimination and persistence.

#### 3.3.6. Exosomes

HBV uses small extracellular vesicles (sEVs) liberated from infected hepatocytes to impair macrophages. These HBV-enriched sEVs affect M1 macrophages and repress production of inflammatory mediators, mitigating innate immune activation [209,210]. This is partly mediated by the increased amounts of microRNAs such as miR-146a and flap endonuclease-1 (FEN-1) within HBV-enriched vesicles, thus promoting viral escape [209]. Moreover, sEVs transfer microRNAs that reduce IL-12 production in macrophages, minimizing antiviral cytokine production [211].

Moreover, sEVs from infected hepatocytes upregulate PD-L1 expression in macrophages, preventing T cell activation through binding to PD−1 in T cells [212].

Exosomes from patients responding to Peg IFN-α treatment and the supernatants of IFN-α-treated macrophage repress HBsAg, HBeAg, HBV DNA, and more importantly covalently closed circular DNA (cccDNA) in HBV-infected cell lines. Peg IFN-α treatment upregulated exosomal miR-193a-5p, miR-25-5p, and miR-574-5p, which could partially inhibit HBV replication, and miR-574-5p that reduced pre-genomic RNA and polymerase RNA levels by binding to the 2750-2757 position of the HBV genomic sequence [213]. Moreover, there have been several reports indicating that certain miRNAs are involved in macrophage polarization [121,214].

## 4. The Role of Liver Macrophages in HCV

### 4.1. Recognition of HCV by Macrophages

The first step of HCV infection of hepatocytes is accomplished through binding with scavenger receptor B1, lipoprotein receptors, tetraspanin CD81 and the tight junction proteins claudin-1 and occludin. Scavenger B1, CD81 and lipoprotein receptors are also expressed in KCs [215,216]. It is unlikely that KCs can support HCV replication, but HCV proteins can be recognized and activate KCs. HCV core and NS3 proteins also activate human BMDMs to secrete TNF-α and IL-10. Depletion of TLR1 or TLR6 from human macrophages significantly affected HCV core and NS3 activation, indicating that these TLR2 co-receptors are involved in stimulation of macrophages by these two HCV proteins [217].

Moreover, TNF-α produced by macrophages increased virus entry into hepatocytes. TNF-α initiated a re-localization of occludin and increased the lateral diffusion of tetraspanin CD81 to support HCV entry [218].

Furthermore, HCV-E2 is recognized by binding to KCs through CD81 [219]. DC-SIGN, a C-type lectin not expressed by hepatocytes also binds HCV on KCs [220,221]. Macrophages stimulated by HCV dsRNA suppressed HCV replication in hepatocytes by producing type I IFN [222].

Phagocytosis of HCV by macrophages leads to increased production of IL-1β and IL-6 and promotion of apoptosis of macrophages [223]. HCV core protein impairs polarization into M1 or M2 phenotypes in chronic HCV patients which can be partially reverted by treatment with direct acting antivirals (DAAs) [224,225]. Similarly, the polarization of M1 macrophages was affected in HCV-infected individuals, which led to significantly decreased IFN-γ expression by CD8+T cells [226]. These findings are in disagreement with another study of in vitro experiments. HCV induced monocyte transformation into macrophages with a mixed M1/M2 cytokine profile and M2 polarization that promoted HSC trans-differentiation via TGF-β [227].

Infectious HCV particles increased monocyte chemoattractant protein 1 (MCP-1) expression in macrophages leading to increased migration of monocytes. IL1β, IL6 and TNFα produced during stimulation of macrophages by HCV were the mediators that induced MCP-1 expression. Long-term HCV incubation induced apoptosis of macrophages [228].

### 4.2. Liver Macrophages in HCV-Disease Pathogenesis

In similarity with HBV, liver macrophages have a dual role in HCV pathogenesis. They either initiate an inflammatory response trying to eliminate the virus, or they are suppressed by viral proteins and allow for the persistence of the disease. Unchecked and protracted inflammatory response may cause liver damage. HCV infection of hepatocytes activated PRRs such as RIG-I and melanoma differentiation-associated protein 5 (MDA-5), as well as induced the IFN response in infected hepatocytes [229,230]. Phagocytosis of HCV by KCs activated the NLRP3 inflammasome leading to the activation of caspase-1 that cleaves pro-IL-1β and pro-IL-18, producing the mature cytokines [231,232,233]. NLRP3 activation of IL-18 induced IFN production in macrophages restricting HCV replication [234]. IL-18 levels were decreased after the acute phase of infection but persisted well above the pre-infection levels throughout chronic infection [235]. In response to hepatocyte injury, increased levels of other inflammatory cytokines, such as TNFα, and IL-12 are produced by KCs, and recruited BMDMs leading to amplification of the inflammation [235,236,237,238,239].

TLRs are critical molecules for the pathophysiology of HCV infection. Upregulation of TLRs on the surface of KCs is associated with the increased production of inflammatory mediators and the increased liver injury. The activation of TLRs on KCs is the mechanism underlying the increased production of type I IFN-β which restricted HCV replication as mentioned above [240]. Serum levels of TLR3 and TLR7 were reduced in patients with HCV compared to healthy controls and correlated with the level of IFN-α [127]. The downregulation of these TLRs on KCs is closely related to immune tolerance and chronicity of HCV. In contrast, increased levels of TLR2 and TLR4 expression were observed in peripheral monocytes of patients with HCV infection and were associated with an increase in circulating TNF-α level and hepatic inflammatory activity [241].

As mentioned before, KCs produced TNF-α after stimulation with core, NS3, NS4, and NS5 HCV-related proteins in amounts comparable to those produced by stimulation with LPS. TLR4 on KCs recognized NS3 and transferred the signaling that activated NF-κB promoting thus production of TNF-α. This production was reduced by 60% after inhibition of TLR4, indicating that TLR4 is not the only pathway of TNF-α secretion [242].

Not only KCs but also other liver cells are involved in the innate immune response against HCV. In vitro, stimulation of human liver cells with TLRs 1–9 ligands for up to 24 h led to secretion of IL-6, TNFα and IL-10 by non-parenchymal cells. However, only supernatants of TLR3-activated KCs, LSECs and HSCs contained type I and type III interferons and an antiviral activity against a sub-genomic hepatitis C virus replicon system. Moreover, TLR3 responsiveness was upregulated in LSECs isolated from HCV-infected patients compared to uninfected controls. It seems therefore, that non-parenchymal cells are initiators of the liver innate immunity [243].

In macrophages, HCV core can bind to the human complement receptor C1qR, abrogating T cell responses [244,245], and repress secretion of the inflammatory cytokine IL-12 [246].

IFN production is also important in the pathogenesis of HCV-induced hepatic injury. Upon HCV entry, the activation of RIG-I and MDA5 signaled the induction of over 300 antiviral Interferon Stimulated Genes (ISGs) in association with the secretion of type I and III interferons [247]. In the hepatocytes where HCV is efficiently replicated, most viral proteins attenuate antiviral innate immunity by inhibiting both RIG-I/MDA5 and IFN-Jak-STAT signaling [248]. In contrast, KCs and BMDMs utilize different PRRs, the TLRs. Sensing PAMPs by TLRs results in the strong cytokine production as described before, which recruits other immune cells to the site of infection to induce the adaptive immune response.

Similar to HBV, the production of IL-1β is important in the pathogenesis of HCV. During chronic HCV numbers of KCs are increased in the liver [249]. HCV drives an immediate but temporary caspase-1 activation to stimulate IL-1β secretion. HCV uptake also initiates a potassium efflux that activates the NLRP3 inflammasome. Viral interference with the NLRP3 inflammasome promotes IL-1β production to trigger pro-inflammatory cytokines and chemokines that are associated with HCV severity [250]. Liver and serum IL-1β levels were increased in patients with chronic HCV as compared to healthy controls [251]. Furthermore, HCV induced KCs to produce cytokines such as IL-6, IL-1β and IFN β which inhibited HCV replication in the HCV replicon model [240,252,253], suggesting that KCs may initiate antiviral activity upon HCV exposure.

Other factors are also implicated in the pathogenesis of HCV. Receptor expressed on myeloid cells 1 (TREM1), which is a member of the immunoglobulin superfamily of receptors, is implicated in systemic inflammation. In vitro incubation of KCs with HCV upregulated TREM1 expression. Moreover, targeting TREM1 with a specific agonist increased HCV-mediated inflammatory responses of macrophages. On the other hand, deletion of TREM1 inhibited inflammation elicited by HCV stimulation. HCV patients were found to express increased levels of TREM1 and higher frequency of TREM1+ CD68+ cells [254].

Pathogenesis of HCV is additionally influenced by alterations in metabolism of macrophages. HCV patients have increased liver macrophages with dysregulated cholesterol metabolism. How HCV interferes with metabolic change in macrophages is not clear. Exposure of macrophages to HCV led to increased lipids and cholesterol and activation of cholesterol-sensing, immunomodulatory liver X receptors (LXRs). Scavenging receptors were responsible for HCV RNA accumulation in macrophages in this in vitro experiment. It was suggested that HCV released from infected hepatocytes stimulated accumulation of lipids and activated LXR in macrophages [255]. LXR activation in macrophages activated genes implicated in the reverse cholesterol transport pathway of cholesterol efflux. It has also anti-inflammatory effects through repression of inflammatory genes [256]. Thus, LXR activation restricts cholesterol formation, enhances cholesterol removal, and reduces innate immunity.

### 4.3. Tolerance and Persistence of HCV

#### 4.3.1. Tolerance and Liver Macrophages

The mechanisms employed by HCV to evade immune clearance are similar to HBV in many aspects.

Non-parenchymal liver cells have a low infection rate in HCV. However, the interplay between the virus and innate immunity is mandatory for the persistence of HCV. HCV affects the immune system through the action of viral proteins including core, non-structural (NS) and envelope proteins that interfere with TLR signaling [127,257,258]. Continuous activation of a particular TLR signaling pathway in KCs may lead to tolerance. Pre-stimulation of the TLR2 signaling pathway with HCV core led to reduced secretion of IL-6 by human antigen-presenting cells after subsequent ligand stimulation of either TLR2 or TLR4. Moreover, TLR ligand-stimulated IL-6 secretion was considerably downregulated in peripheral monocytes of HCV patients, compared with those of healthy controls indicating that chronic stimulation with HCV core protein leads to reduction in TLR-mediated innate immunity and persistence of HCV infection [259].

The HCV NS proteins may also impair innate immunity. NS3 can mimic the action of TGF-β to promote fibrosis through binding to TGF-β type 1 receptor (TβR1) [260]. Increased production of TNFα during HCV intensifies this interaction. NS3 also inhibits the TLR3-induced antiviral response. Taken together, these actions of NS3 promote tolerance [261,262]. NS3 also binds to TLR2 to initiate heterodimerization with TLR1/6 increasing the expression of IL-8, IL-10, and TNF-α [230]. Furthermore, NS3 and NS4A protein inhibited the expression of IFN-α/β/γ and the chemokines CCL-5, CXCL-8, and CXCL-10 in macrophages [263]. The NS3/4A complex can also restrict innate immunity by blocking the antiviral response generated by RIG1 upon detection of dsRNA [264]. This complex was shown to ablate RIG1-1 induced IRF3 and NF-κB activation leading to inhibition of IFN-β production [265,266].

Persistence of HCV is also mediated by the interference of HCV proteins with the M1/M2 polarization of macrophages. HCV core inhibited polarization to either M1 or M2 phenotypes impairing STAT signaling pathway. In addition, HCV core reduced phagocytosis of M1 and M2 cells resulting in dysfunction of both M1 and M2 macrophages in chronic HCV patients [225].

Subtype M2 alterations caused by HCV also mediate HCV persistence. HCV prevented M2a, M2b and M2c subtype polarization leading to defective phagocytosis in patients with HCV. HCV core was responsible for these alterations including the repression of phagocytosis. This defect was reverted by an anti-TLR2 antibody [267]. High HCV levels not only repressed the differentiation into M1 macrophages, but also shifted macrophage polarization toward a tolerogenic state [268]. Interestingly, HCV induced polarization of macrophages expressing a mixed M1/M2 cytokine profile but an M2 surface phenotype that induce HSCs activation via TGF-β [227].

The divergent results of HCV on macrophage polarization are difficult to explain. Again, direct comparisons cannot be made as there are many parameters that may influence the results. Data are provided by either in vitro models or clinical studies. Incubation times vary in experimental conditions, and the macrophage subset analyzed each time is difficult to be characterized in view of the various subsets recently described. In clinical studies disease stage, viral loads and different genotypes may play a role in the results in addition to the difficulties to characterize the exact macrophage subtype.

Table 2 summarizes the main effects of viral proteins on macrophages.

#### 4.3.2. Implication of TLRs in Tolerance and Persistence

The importance of TLRs in HCV tolerance and persistence was indicated above. Further evidence supports this. The decrease in TLRs on KCs is intimately associated with chronicity of HCV. It has been demonstrated that HCV upregulated miR-758 levels and reduced TLR3/TLR7 expression impairing the innate immune response [275]. Experimental studies demonstrated that TLR7 mRNA was reduced in hepatocytes infected with HCV, while after clearance of the infection TLR7 expression returned to previous levels [276]. TLR7/8 were also described as mediators of M2 polarization during HCV infection. HCV ssRNA and other TLR7/8 ligands increased M2 polarization and generation of circulating fibrocytes [277].

#### 4.3.3. Μacrophage Immune Checkpoints and Virus Persistence

There are other mediators of HCV chronicity. Interaction of viral proteins with immune checkpoints or IL-10 production are important in that respect. KCs induced PDL1 and PD-L2 during HCV infection leading to repression of T cell response [175,278,279]. PD-1 is expressed on HCV-specific CD8+ T cells of chronic HCV patients. Inhibition of the PD-1/PD-L1 interaction restored T cell activity [280,281]. Liver HCV-specific CD8+ T cells expressing high amounts of PD-1 were functionally exhausted, but inhibition of PD-1 and CTLA-4 reversed exhaustion [271,282]. The regulation of PD-L1 has not been clarified, but IFN-a, β, and γ seem to promote PD-L1 expression [283,284,285]. More specifically, it is the stimulation by the HCV core protein that induces PD-L1 expression by KCs. HCV core inhibited TLR3-induced production of IFN-α and β. Inhibition of phosphoinositide 3 kinase by HCV core also abrogated the TLR3-specific induction of the cytotoxic molecule TRAIL [272].

Galectins are glycan-binding proteins implicated in both innate and adaptive immunity. Galectin-9 is the ligand for the T cell immunoglobulin domain and mucin domain protein 3 (Tim-3) [286]. An increase in galectin-9 has been reported in chronic HCV patients. KCs are the source of galectin-9, and its interaction with Tim-3 leads to apoptosis of HCV-specific cytotoxic T lymphocytes [287]. Both PD-1 and Tim-3 are increased on cytotoxic T cells from either the liver tissue or from the peripheral blood and are associated with T cell exhaustion in HCV hepatitis [280,288]. Tim-3 is upregulated on virus-specific T cells of HCV-infected patients, and Tim-3 inhibition revokes exhaustion and restores CD4+ and CD8+ T cell function in chronic HCV infection [289].

Galectin-9 also initiated the proliferation of regulatory T cells [290]. Exosomes released from HCV-infected hepatocytes stimulate galectin-9 production by cultured monocytes, and circulating monocytes have the highest Gal-9 amounts in chronic HCV patients [291]. Galectin-9 also modulated NK cells impairing cytotoxicity and cytokine secretion [292]. Taken together, current evidence indicates that galectin-9 production by hepatic macrophages maintains chronic HCV infection through regulation of NK- and T cell responses.

#### 4.3.4. IL-10 Production and M1/M2 Balance

IL-10 is another mechanism by which HCV establishes its persistence. Increased levels of IL-10 mRNA expression were demonstrated in monocytes from patients with chronic HCV [293], while plasma levels of IL-10 were significantly increased in chronic HCV compared to healthy controls [278,294]. HCV core induced IL-10 production by KCs leading to repressed production of pro-inflammatory mediators [217,270,272]. IL-10 also reduced major histocompatibility complex (MHC) class II molecules and co-stimulatory molecules, so communication between KCs and NK cells was impaired [70,295]. Antigen-presenting function of KCs was impaired by IL-10 [296,297,298]. IL-10 can also repress the action of T cells against the virus [146].

The critical significance of the interaction between NK and macrophage for the elimination of HCV is suggested by the various methods used by the virus to modulate NK cell and macrophage coordination. Reduced IFN-γ production by NK cells shifts the differentiation of macrophages toward an M2 phenotype and an immunosuppressive environment that favors HCV persistence [273].

**Figure 3:**
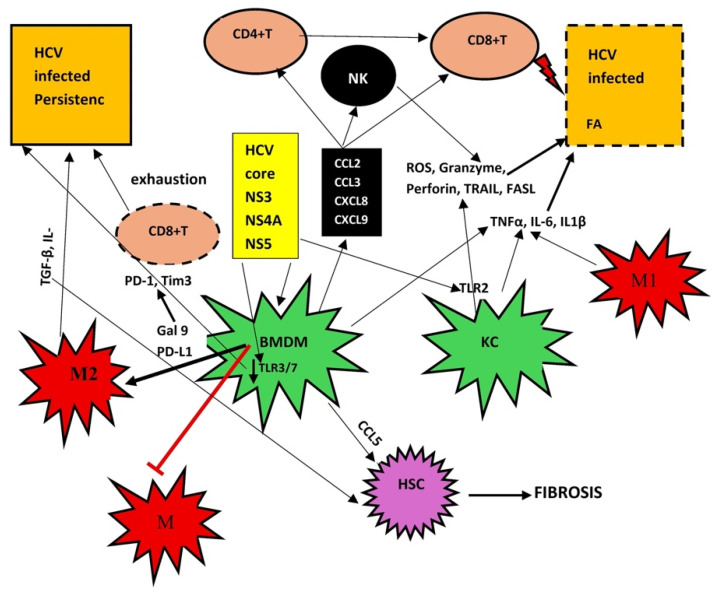
The role of macrophages in HCV inflammation and persistence. Kupffer cells and bone marrow-derived macrophages recognize HCV proteins and produce pro-inflammatory cytokines that can damage hepatocytes and molecules such as TRAIL and FASL that can lead hepatocytes to apoptosis. Moreover, they produce chemokines that attract other immune cells that also attack infected hepatocytes in a way similar to HBV. Viral proteins block M1 polarization and promote the M2 phenotype. As a result, a tolerogenic environment driven by IL-10 and TGF-β is established that leads to HCV immune evasion. This is further supported by the exhaustion of HCV specific CD8+ effector T cells through the galectin-9-Tim-3 and the PD-1/PDL-1 checkpoint systems that are upregulated by viral proteins. The same proteins reduce the expression of TLR3/TLR7 receptors enhancing also virus persistence. For further details see text.

Figure 3 diagrammatically presents the role of macrophages in HCV elimination and persistence.

#### 4.3.5. Exosomes

HCV also uses extracellular vesicles (EVs) for immune escape and persistence of infection [299]. Exosomes secreted by infected hepatocytes may suppress the function of KCs and macrophages [300]. On the other hand, EVs secreted by activated macrophages or LSECs contain type I/III interferons or antiviral miRNAs that repress HCV replication, indicating that exosomes may have opposite roles according to their source [301].

In summary, HCV infection has a dual effect that can be both pro-inflammatory and tolerogenic. The dual role of HCV proteins is better exemplified by data on the effects of NS5A which can bind to TLR4, to increase IFN-β, TNF-α, and IL-18 production demonstrating thus a pro-inflammatory/pro-fibrotic profile [230]. NS5A can also promote inflammation by increasing NF-κB and STAT-3 activation through induction of ROS and by increasing Ca2+ signaling [302]. On the other hand, NS5A binding to TLR4 may instead initiate p38- and PI3K-dependent IL-10 and TGF-β secretion inhibiting IL-12 production [303]. It also inhibits NKG2D expression on NK cells preventing IFN-γ secretion and destruction of HCV-infected cells [274]. Taken together, the evidence indicates that HCV proteins modulate several pathways of innate immunity to avoid elimination, while at the same time may promote liver injury.

Finally, increased iron of macrophages may lead to enhanced HCV replication through reversed ferritin flow. It was shown that viral transmission from infected macrophages to uninfected hepatoma cells was initiated by iron. HCV possibly increased intracellular iron sequestration through hepcidin and intercellular iron mobilization via ferritin [304].

## 5. The Role of Macrophages in Virally Induced Fibrosis and Cirrhosis

During the development of viral liver fibrosis, pro-fibrotic factors, such as TGFβ, CTGF, ROS, and PDGF from infected hepatocytes, activate KCs, BMDMs, LSECs, cholangiocytes and HSCs [305,306,307]. During viral hepatitis, KCs produce cytotoxic molecules, like TRAIL, Fas-ligand, granzyme B, perforin, and ROS, that act as executioners for infected as well as non-infected neighboring hepatocytes. Granzyme B and perforin expression by KCs was upregulated in both chronic HBV and HCV patients [71,308].

Damaged hepatocytes release apoptotic bodies that are phagocytosed by KCs and HSCs. Transformation of HSCs into myofibroblasts [309,310] led to extracellular matrix deposition accompanied by decreased metalloproteases (MMPs) and increased tissue inhibitors of MMPs (TIMPs), originating from macrophages and HSCs [311,312]. Recently, cadherin-11 was identified as an important mediator of liver fibrosis [313]. Interestingly, this protein was produced by injured hepatocytes, HCSs and macrophages. In addition, both HBV and HCV increase fibrosis via a TGF-β1-induced octamer binding transcription factor 4 (OCT4) /Nanog-dependent pathway [314].

KCs participate in virally induced fibrosis through additional mechanisms. They promote the cross-linking of collagen fibrils via lysyl oxidase-like 2 (LOXL2) [315,316]. Moreover, KCs promote HSC survival producing growth factors such as PDGF and CCL5 [317,318,319]. On the other hand, KCs have a dual role in fibrosis as they are implicated in matrix degradation through the production of MM9 [320]. In that respect, anti-fibrotic effects have been reported after infusion of KCs [321]. However, experimental evidence indicates that these activities are partially due to KCs but mostly depend on recruitment of BMDMs [32,322] as a result of liver injury [249,323].

During fibrosis resolution, evidence from murine models of liver has shown that reduction recruited Ly6C^high^ macrophages decreased HSC transformation and matrix deposition, while reduction in Ly6C^low^ macrophages during the resolution phase impairs matrix degradation. Ly6C ^low^ macrophages represented the main matrix metalloproteinase expressing subset [324]. The switching from Ly6C^high^ to Ly6C^low^ macrophages follows the phagocytosis of damaged cells, a process called efferocytosis.

In human cirrhosis, phagocytosis and anti-bacterial activity of KCs are diminished [325] increasing vulnerability of patients to bacterial infections [326]. KCs may be implicated in portal venous pressure increase producing vasoconstrictors such as cysteinyl leukotrienes [327].

The conflicting results in human KCs studies might reflect the extreme heterogeneity and plasticity of KCs as the influence of BMDMs could not be assessed, particularly in earlier studies. An extensive study of more than 100,000 single human cells reported that a TREM2+ CD9+ subset of macrophages (SAM cells) that derive from BMDMs and certain pathways such as NFRSF12A, PDGFR and NOTCH signaling are involved in fibrogenesis [50]. These findings have been confirmed by another study [328]. The exact role of SAMs in viral diseases has not been clarified [329].

The function of TREM1 and TREM2 is important in inflammation and fibrosis. Early in liver diseases, the function of TREM1 is prevailing but gradually the biological function of TREM2 becomes dominant. In acute inflammation, TREM1 expressed on KCs upregulates production of cytokines and recruits BMDMs enhancing inflammation, while TREM2 expressed on KCs represses inflammation. During fibrosis, TREM1 of KCs initiates HSCs transformation. TREM2 expressed on macrophages favors the accumulation of collagen fibrils [330,331].

In areas of fibrosis both M1 and M2 macrophages are present suggesting that both are involved in fibrosis. During fibrosis resolution, M1 polarized macrophages prevail with a concomitant reduction in M2 suggesting their importance in matrix degradation [332]. Similarly, M2 macrophage activation was associated with liver fibrosis during chronic HCV infection in the livers of patients, while direct acting antiviral therapy attenuated M2 macrophage activation and associated liver fibrosis [333].

The granulocyte-macrophage colony-stimulating factor (GM-CSF) also participates in the progression of virally induced fibrosis. In patients with chronic HCV, serum GM-CSF were correlated with fibrosis and serum viral titers. Moreover, anti-GM-CSF neutralizing antibody minimized hepatic CD206+ macrophages and ameliorated fibrosis in HBV-infected humanized mice [334]. However, it should be stressed that the concept of the clear distinction of M1 and M2 macrophage polarization was mostly based in vitro observations of cultured monocytes. Such distinction of M1 and M2 macrophage phenotypes is blurred in in vivo observations, where M1 and M2 markers may co-exist on the same macrophages at least in other fibrotic diseases [335,336]. Whether this is relevant for liver fibrosis and cirrhosis remains to be proved.

Further evidence that macrophages are implicated in liver fibrosis was recently provided. Serum sCD163 levels were considerably increased in patients with HCV as compared to healthy controls and were correlated with liver fibrosis. Serum sCD163 was a reflection of hepatic CD163 expressing macrophages in the liver sections from patients [337].

In addition, exosomes regulate the progression of fibrosis being important component of the communication network between cells. Depending on the load of transported miRs, exosomes may enhance or prevent liver fibrosis and macrophage differentiation [338,339,340]. Macrophage exosomes carrying miR-103-3p promoted HSCs trans-differentiation [341]. Moreover, LPS stimulation of the same macrophage cell line released exosomes rich in miR-155-5p or miR500 that enhanced ROS and collagen synthesis by HSCs [342]. On the contrary, exosomes with miR-411-5p originating from M2 macrophages prevented HSC transformation [343].

Hepatocyte cytotoxicity due to lipid overload results in exosome production rich in miR-192-5p that initiated M1 macrophages polarization [344]. Overexpression of lipid-induced death receptor 5 in the surface of injured hepatocytes led to the release of exosomes that also enhanced M1 polarization [345]. Hepatocytes overloaded with cholesterol released exosomes rich in miR-122-5p that induced the M1 phenotype [346]. Furthermore, exosomes released by transformed HSCs initiated a pro-inflammatory polarization of macrophages [347], while miR-148a containing exosomes originating from mesenchymal stem cells switched macrophages from M1 to M2 polarization [348].

Although these data were generated from MASLD models, it is tempting to assume that they are also applicable in chronic HCV, as hepatocyte lipid accumulation is a common finding in this disease.

In summary, current evidence indicates that exosomes with different content of miRs are implicated in liver fibrosis acting either directly activating HSC or indirectly through pro-inflammatory polarization of macrophages [349].

## 6. Macrophages in Virally Induced Acute-on-Chronic Liver Failure (ACLF)

Acute-on-chronic liver failure (ACLF) is a complex clinical syndrome associated with high mortality. It is characterized by strong systemic inflammation superimposed on a pre-existing chronic liver disease and is accompanied by organ failure. Most cases of ACLF in Asia Pacific and Africa are connected to HBV infection while in the West they are associated with either alcohol or drug hepatotoxicity [350].

As mentioned before, initiation of inflammation occurs upon activation of KCs through binding of PAMPS and DAMPs to their PRRs. Activated KCs recruit BMDMs to the liver to augment inflammation [351,352]. Initially, IL-33 acting as a danger-associated molecular pattern (DAMP) enhanced macrophage-induced inflammation through ERK1/2 activation during HBV-related ACLF without compromising their phagocytic activity [351]. In contrast, the later stages of ACLF were characterized by immunosuppression, where the dysfunction of macrophages was evident by the decrease in HLA-DR expression. As a result, antigen presentation was impaired, and the production of inflammatory cytokines was reduced [353]. At later stages low HLA-DR expression was negatively associated with prothrombin time [354].

Another marker of immunosuppression is the increase in MER tyrosine kinase (MERTK). The MERTK overexpression was correlated with ACLF immunosuppression and disease severity [355]. The rise in immunosuppressive CD14+HLA-DR-myeloid-derived suppressor cells repressed T cell function, attenuated anti-microbial innate immune responses and were responsible for secondary infection and prognosis [356,357].

Macrophage metabolism is also affected in ACLF. In the leukocytes of ACLF patients, mitochondrial function analysis uncovered break-points in the TCA cycle at the isocitrate dehydrogenase and succinate dehydrogenase level, which were compensated with reactions involving glutaminolysis. The metabolites of the TCA cycle can modulate macrophages. A low ratio of α-ketoglutarate/succinic acid switches macrophage polarization toward the M1 phenotype [358].

Moreover, mitochondrial dysregulation can lead to increased aerobic glycolysis promoting lactic acid production by macrophages during inflammation [359] that restricts inflammation and inhibits recruitment of BMDMs [360,361]. Omega-3 polyunsaturated fatty acids were significantly decreased in patients with ACLF, although total fatty acid levels were considerably raised [362,363,364]. Circulating saturated fatty acids lead to amplification of inflammation by increasing the responsiveness of immune cells to TLR agonists. This is reversed by increased intake of linoleic acid through upregulation of prostaglandin E2 production that in turn reduces macrophage production of inflammatory cytokines [365,366].

Macrophages show different phenotypes and functional characteristics in HBV-related ACLF in comparison with ACLF of other causes, possibly due to different tissue microenvironments, that ultimately affects the clinical outcome of ACLF. This discrepancy cannot be explained at the moment [367].

Clinically, plasma levels of the macrophage biomarkers sCD163 and CD206 are closely associated with the severity of ACLF [368]. Proteolytic cleavage of CD163 from the surface of macrophages is mainly responsible for the increased plasma levels [53].

STING is also implicated in ACLF. The result of STING activation in HBV-related ACLF was studied in a murine model. Early in STING activation autophagy flux was enhanced, and inflammation and liver damage were ameliorated. By contrast, at the late stage of STING activation macrophages were switched toward the M1 phenotype, aggravating inflammation and liver damage [369].

## 7. Macrophage Targeting in the Treatment of HBV

The progress in the contribution of KCs and liver macrophages in the pathogenesis of HBV-and HCV-related liver disease that has been made during recent years led to efforts to target macrophages for treatment of chronic viral disease. Direct acting antivirals (DAAs) with their high success rate in viral elimination possibly indicate that development of macrophage-based drugs may not be necessary for HCV treatment, which may not be true.

Treatment with DAAs effectively targets more prevalent strains (such as 1a, 1b, and 3a) but non-epidemic subtypes may be resistant. Moreover, some strains are inherently resistant to currently available DAAs due to the presence of natural polymorphisms at resistance-associated substitution positions. As mentioned before, the host’s immune system identifies some structural and NS HCV proteins through receptor retinoic acid-inducible gene-I (RIG-I)-like receptors and toll-like receptors (TLRs) in Kupffer cells, that trigger the production of antiviral cytokines such as interferons IFNs. IFN-stimulated genes (ISGs) inhibit HCV replication and activate natural killer (NK) cells. Resistance to ISGs enable HCV variants to escape selection pressures [370].

Moreover, HCV has been shown to induce extensive long-lasting epigenetic modifications in the host genome. Thus, H3K27me3 (histone 3 lysine 27 trimethylation) gain throughout the TLR3 gene locus and loss of H3K27ac (histone 3 lysine 27 acetylation) at the same promoter lead to TLR3 silencing, anti-apoptosis and immune response suppression, and silencing of key tumor suppressor genes. Therefore, even with successful treatment, the risk of liver cancer persists for many patients [371].

By contrast, available treatment modalities are less effective in eradicating the HBV from hepatocytes due to the persistence of the covalently closed circular DNA (cccDNA), HBV DNA integration into the cellular genome, and viral negative impact on the host immune system. Two types of treatment are approved for HBV infections. Oral nucleos(t)ides analogs (NAs) are the first-line anti-HBV treatment worldwide. NAs reduce viral load by inhibiting HBV polymerase activity. NAs therapy can suppress viremia to clinically undetectable levels in up to 76% of Hepatitis B e antigen (HBeAg)-positive patients and up to 93% of HBeAg-negative patients after one year of treatment. However, their functional cure rate (undetectable HBsAg) remains <10% over long-term follow-up due to insufficient efficacy and low resistance barriers. Another treatment strategy is immunomodulatory therapy enhancing immune cell recognition and killing of HBV-infected cells, thereby breaking immune tolerance. This approach achieves ≤20% functional cure rates but is frequently associated with serious adverse reactions [372].

Therefore, therapeutic agents are in clinical development or in early translational approaches in an effort to maximize treatment success in HBV.

CTLA-4, IL-10, and TGF-β produced by Kupffer cells inhibited dendritic cell (DC) maturation, and the interaction between Tregs and DCs reduced the activation of effector T cells by DC. HBsAg-specific Tregs mediated follicular helper T cell (TFH)-dependent HBsAb dysregulation by limiting the differentiation of HBsAg-specific TFH cells, resulting in reduced production of HBsAb against HBV-infected hepatocytes. Removal of Tregs or blocking CTLA-4- and HBsAg-specific TFH cells in patients with CHB restored their ability to clear HBV, a fact that can be used as a target for CHB treatment [373,374].

Depletion of KCs by CLD liposomes enhanced Ly6C+ monocyte recruitment and accelerated HBV clearance in mice. These two cell types play an essential role in determining HBV clearance/tolerance as mentioned before. Manipulation of these cells is a promising tool for immunotherapy of HBV-related liver diseases [125].

Toll-like receptors (TLRs) enhance antiviral immunity primarily through innate immune activation. TLR8 agonism enhanced HBV-specific B cell responses in chronic HBV patients by increasing monocyte-mediated TFH function, and functional cure [375].

In addition to TLRs immune liver cells express other pattern recognition receptors such as RIG I/MDA5, which induce innate immunity through sensing of pathPAMPs as mentioned above. TLR/RIG I agonists suppress HBV replication in vitro and in vivo and are investigated in clinical trials. On the other hand, HBV-specific immune responses could be induced by therapeutic vaccines, including protein (HBsAg/preS and HBcAg), DNA, and viral vector-based vaccines [376].

Recently a phase II trial was contacted using Selgantolimod, which is an oral TLR8 agonist in HBV patients already under oral nucleos(t)ide analog treatment. Selgantolimod administration was associated with modest reductions in HBsAg and HBeAg levels, but only rare loss of HBsAg (5%) or HBeAg (16%) [377].

A TLR7 agonist (GS-9620) has also been tried in a phase 1b trial, but the results were not particularly encouraging [378].

Shifting the interest to adaptive immunity, co-stimulatory signals are important for an effective T cell response. The percentage of T cells expressing the checkpoint OX40 was reduced in patients with chronic hepatitis B compared to healthy adults and was negatively correlated with serum viral load. Moreover, the activation of OX40 suppressed HBV replication in a CD8+ T cell-dependent manner [379].

Preclinical studies that may lead to clinical trials include the selective targeting of CD163 + macrophages [380] or the silencing of PD-L1 in the liver using small interfering RNA (siRNA) in combination with a therapeutic vaccination scheme (TherVacB). In mice experiments, there was an improved function of HBV-specific CD8+ T cells [200].

Table 3 presents certain therapeutic efforts to modulate liver macrophages in chronic HBv. Most studies are in phase I or II. The subject has been very recently reviewed [184].

## 8. Conclusions

Kupffer cells and recruited BMDMs are heavily implicated in the pathophysiology of HBV and HCV-related hepatitis. Viral protein such as HCV core and NS5a in HCV and HBsAg HBcAg and HBeAg in HBV initially drive an inflammatory response through NF-κB and NLRP3 inflammasome activation. TNF-α, IL-6, and IL-1β are the main inflammatory cytokines produced by activated liver macrophages. Anti-inflammatory effects are usually late after stimulation with HBx, HBeAg for HBV, and NS3/4A, core, and NS2 for HCV. The reason for these contradictory effects has not been clarified, but it is possible to reflect the extreme plasticity of liver macrophages. Viral proteins also mediate the polarization of macrophages either toward an M1 inflammatory phenotype, or toward an M2 anti-inflammatory immunosuppressive type. Viral proteins also drive macrophages to develop a tolerogenic profile that promotes immune evasion for the virus leading to persistence of the infection. Infiltrating BMDM exposed to some of the pro-inflammatory viral proteins are the primary inflammatory cells, while KCs after an initial pro-inflammatory state promote the production of anti-inflammatory cytokines and fibrosis. Current data show that these changes are mediated through TLR signaling. HBcAg and HCV structural proteins drive pro-inflammatory cytokine production by activating TLR2 but other TLRs are also implicated. the way viral infections affect different populations of liver macrophages require additional investigation for better definition of the macrophage clusters involved. A better discrimination of the many macrophage subsets involved is necessary using the currently available single-cell techniques such as scRNA-seq that may change our perception of the role of liver macrophages. It should be noted that macrophages of a distinct phenotype may produce cytokines characteristic of another phenotype. These contradictory findings probably suggest that the ultimate result is the consequence of the type or the degree to which receptors are stimulated. In the future, there is clearly an urgent need for human longitudinal studies, sex/age-stratified studies, and validation of recent findings such as the persistence of iMacs after cure in larger cohorts. Moreover, a better distinction will help to devise better approaches of treatment modalities that may be used in HBV elimination.

## Figures and Tables

**Table 1:** Heterogeneity of liver macrophages.

Murine	Function	Ref.
Kupffer cells (KCs) CLEF4F+, VSIG4+, CLEC2+, FOLR2+, TLRs, RLRs	Liver tolerance, fibrosis Pro-inflammatory	[29]
Ly-6C^high^	Pro-inflammatory	[33,34]
Ly-6C^low^	Restoration	[33,34]
EmKCs	Pro-inflammatory	[35,36,37,38]
moKCs Marco+, Tim4+	Phagocytosis	[35,36,37,38]
KC1: CD206^low^, ESAM^-ve^	Protection from drug-induced injury	[45]
KC2: CD206^high^, ESAM^+ve^	Fatty acid metabolism	[43]
KC2: CD36+ve	Regulation of the obesity-related oxidative stress	[43]
KC2:CD36-ve	Attenuation of inflammation	[44]
LAM:Trem2+ve, CD9+ve	Inflammatory	[61,62,63]
**Human**	**Function**	**Ref.**
CD14^high^, CD16^-ve^	Correspond to Ly-6C^high^	[34]
CD14^-ve,^ CD16^high^	Correspond to Ly-6C^low^	[34]
CD163+ve	Removal of Hb–haptoglobin complex	[53,66]
CD32^high^	Endocytosis–immune suppression	[54]
CD32^low^	Inflammation–anti-microbial activity	[54]
TREM2+ve, CD9+ve (SAM)	Scar-associated macrophages	[56,57]
MERTK+ve	Protective in acute liver failure	[64]
CD49a	Production of IL-10	[55]
CD68+ve, MARCO+ve, TIMD4+ve	Immune tolerance	[50,51,52]
CD68+ve, MARCO-ve, TIMD4-ve	Pro-inflammatory	[50,51,52]
iMAC CD40, CD16	Inflammatory	[65]

**Table 2:** Effects of viral proteins on macrophages.

HBV Proteins	Effect	Mechanism	Ref.
HBx	Inhibition of antiviral effects	Reduction in IFN production. Impairs mitochondrial antiviral signaling.	[188]
HBeAg	Inhibition of NLP3 inflammasome reduction in IL-1β secretion	Inhibition of NF-kB phosphorylation. Reduction in ROS production.	[159]
**OR** HBeAg	Favors inflammation	Increase in TNFα, IL-6 production.	[190]
HBV polymerase	Inhibition of IFN production	Prevention of STING-dependent DNA sensing.	[189]
HBsAg	Induction of pro-inflammatory cytokines	Interaction with mannose receptors.	[124,269]
**OR** HBsAg	Inhibition of M1 cytokines but not IL-10	Impairs TLR2/TLR4 signaling.	[191,192,193,194]
HBcAg	Favors viral elimination	Increase expression of TLR2 in M2 macros.	[125,131]
**OR** HBcAg	Promotion of HBV persistence	Increases PD-1 in CD4 T cells. Increases PD-L1 in macros. Increases T cell exhaustion.	[195]
HCV Proteins			
HCV core	Viral persistence	Inhibition of IFN production. Induction of PD-L1 expression in KCs. Induction of IL-10.	[217,270,271,272]
NS5A	Viral persistence	IL-10 induction. IL-12 reduction.	[273,274]
OR NS5A	Pro-inflammatory	Bind to TLR2. Increase TNFα, IL-18.	[230]
NS3, NS4, NS5	Pro-inflammatory	NS3 binds to TLR2/TLR4 activating NF-kB.	[230,242]
NS3	Promotion of fibrosis	Mimics the action of TGF-β through binding to TGF-β type 1 receptor.	[260]

**Table 3:** Therapeutic strategies targeting macrophages in chronic HBV infection.

Therapeutics	Mechanism of Action	Ref.
Vesatolimod (GS-9620)	TLR7 agonist	[381]
RO6870868	TLR7 agonist	[382]
JNJ-64794964	TLR7 agonist	[383]
GS-9688	TLR8 agonist	[376]
SB9200	RIG-I agonist	[376]
GS-9992	RIG-I agonist	[376]
Selgantolimod	TLR8 agonist	[174,377]
Nivolumab	Anti-PD-1	[384]
Pexidartinib	CSF-1/CSF-1R Inhibitor	[385]

## Data Availability

No new data were created in this study. Data sharing is not applicable to this article.
